# European Policies to Promote Children’s Rights and Combat Child Poverty

**DOI:** 10.3390/ijerph14080837

**Published:** 2017-07-26

**Authors:** Mona Sandbæk

**Affiliations:** Faculty of Social Sciences, Oslo and Akershus University College of Applied Sciences, 0130 Oslo, Norway; mona.sandbak@hioa.no

**Keywords:** children’s rights, child poverty, positive parenting, support and provision for parents, progressive universalism

## Abstract

The upbringing of children relies heavily on shared responsibilities between parents and society. The Council of Europe Recommendation (2006) 19 on Policy to Support Positive Parenting and the European Commission Recommendation (2013) Investing in Children: Breaking the Cycle of Disadvantage, both aim at supporting parents to care and provide for their children in accordance with the UN Convention on the Rights of the Child. By means of a document analysis this article examines what kind of parental practices and provision to parents the recommendations suggest to safeguard children’s rights in the family. Three findings are highlighted: first, both recommendations reflect a commitment to respecting children’s rights while at the same time acknowledging parents as children’s primary caregivers. Second, both recognize parents’ rights to work, while also recognizing the necessity of adequate income support if work is not available or income too low. Third, adequate resources are defined as a combination of universal policies and services, which guarantee a minimum level for all, and targeted measures reaching out to the most disadvantaged. The recommendations’ emphasis on children and parents as partners and on the families’ economic situations are valuable for future development of family and child policy and support programs.

## 1. Introduction

The U.N. Convention on the Rights of the Child (UNCRC) has become a cornerstone in promoting a view of children as citizens and holders of individual rights, and their interests as paramount. According to Article 3, the best interest of the child shall be a primary concern. Most often, although not always, parents play an important role in safeguarding children’s rights, which is also acknowledged in the UNCRC. The general intention of the Convention is not to promote a view of children as individuals separate from their families. On the contrary, according to the preamble, caring family environments are best suited to facilitate children’s rights [1]. The Convention emphazises parents’ core role in children’s upbringing and the vital role states play in providing them with appropriate support [2].

This article focuses on how to support parents in implementing children’s rights in everyday life with a particular emphasis on the three Ps; participation, protection and provision. Children are entitled to protection as well as participation. While protection shed light on children as vulnerable, participation often portray children as competent. These are, however, not competing concepts. Adults must safeguard children’s rights to participate, as well as their rights to care and belonging [3]. Article 27 concerns the third P—Provision—and children’s rights to a decent standard of living. The main responsibility to provide for children lies with their parents. If they are not able to do so, state parties are expected to help them. Enabling parents to provide for their children has importance for the well-being of each individual child, but also represents a vital part of providing all children with the equal opportunities called for by the Convention. Unfortunately, unequal living conditions not only still exist, but there are signs of growing economic inequality, including in Europe and the Nordic countries [4,5]. The increasing inequality makes it even more crucial to safeguard the material living conditions of children and their families.

Promoting children’s rights and supporting parents may seem easy to combine, but research indicates that there is a risk of giving priority to children without considering how to enable their parents to support them, or conversely, overlooking children’s interests and focusing on the parents [6]. For example, it has repeatedly been demonstrated that social services rarely consult children and young people even in matters that concern them [7,8]. On the other hand, children may receive economic support to participate in activities, while their parents, living in deep poverty, receive no such benefits, and may be blamed for failing to meet the expected standards for their children [6]. To find a balance between safeguarding children’s rights and strengthening the families’ capacities to care for them, it is an imperative for a society to facilitate parental commitment to children’s rights. As pointed out by the Danish researcher Kampmann ([9], p. 18) “We cannot think of children in new ways without also altering our understanding of what it is to be a parent”. UNICEF [10] argues that the link between children’s well-being and the well-being of their parents makes it of utmost importance to support families in fulfilling the rights of children.

International conventions set standards and establish commitments for governments, while recommendations provide guidelines for developing and implementing policies. To this end, the Council of Europe and the European Commission are two rather different organisations. The Council of Europe is a pan-European human rights organization, with 47 member states, founded in 1949, whereas the scope of the European Union is an economic and political union between 28 European countries, created in 1958. Both play pivotal roles in setting standards related to social rights and social policy issues in Europe. Their member states decide their own social policy, but recommendations of both organisations draw up guidelines for policy makers on the national levels, which also may be instrumental for professionals, organisations and others. In this article, two such recommendations are analysed: (1) the Council of Europe Recommendation (2006) 19 on Policy to Support Positive Parenting and (2) the European Commission Recommendation (2013) Investing in Children, Breaking the Cycle of Disadvantage. Both aim at strengthening children’s rights and supporting parents in implementing their rights. The article examines what these recommendations add to family policies and the field of support to families with regard to the research questions: (1) What kind of parental practices do the recommendations suggest to implement children’s rights in the family, with a particular emphasis on protection and participation? (2) What kind of support and provision do the recommendations suggest to secure children’s rights to a decent standard of living?

## 2. Methods and Materials

The two European recommendations are subject to a document analysis. The first document, the Council of Europe Recommendation Rec (2006) 19 on Policy to Support Positive Parenting [11] consists of two parts. An introduction places the recommendation in the context of other Council of Europe documents, followed by an appendix containing definitions, principles, objectives and core elements. The explanatory report with details constitutes the second part, including two appendices “Key messages for parents” and “Guidelines for professionals”. The second document, the European Commission Recommendation of 20 February 2013, Investing in Children: Breaking the Cycle of Disadvantage [12] is also quite comprehensive. The first part states the overall aim, while the subsequent articles present the principles, integrated strategies and other relevant EU instruments. The two recommendations cover wider areas than the article, which limits itself to explore the research questions using a documentary analysis.

Content analysis has been an accepted method, with the strength of narrowing down the text, but also criticized for being unable to capture the contexts within which a written text has meaning. It has also been pointed to the uncertainty concerning how documentary research can throw light on the relationship between what people say and what happens in reality [13,14]. Still today, according to Mik-Meyer [15] relatively few sociologists have addressed analysis of documents from a methodological perspective. When documents have status as empirical material, she encourages researchers to be aware of the external relationships that characterise the production—as well as the consumption—process. The author relates document analysis to concepts such as institution/context, interaction/process and standardising/facts. In this article, the institution/context dimension is highly relevant, drawing the attention to the institutional contexts where the texts are produced as well as to the member states where the content is eventually put into practice.

The documents analysed in this article, are closely linked to one of the functions of the Council of Europe and the European Union; namely to issue policy recommendations to member states. Although not being legally binding and being within the competence of member states to decide, the two organisations provide guidelines within the field of social policy. The aim of this article is, by means of a document analysis, to examine the recommended policies in the field of children’s rights. To fit into the different contexts of the member states, the recommendations are not standardised or unambiguous, which also justifies applying broad definitions of the concepts. The key concepts in the analysis are parents, parental practices, support and provision to parents. Rather than narrowing down the concepts, the intention is to grasp the variety behind each of them. The concept “parents” is used in accordance with the definition in Council of Europe Recommendation 2006 (19) as persons having parental authority or responsibility. “Parental practices” refer, in this context, to practices regarded as enhancing children’s rights to protection, participation and provision in the family. The term “support” covers a variety of policies and measures comprising informal, semi-formal and formal services to children and parents, and to facilitate combining work and family-life. “Provision” includes redistribution, universal policies and services as well as targeted measures.

## 3. Literature Review

### 3.1. Family and Parenting Support

To provide a background for the analyses of the two recommendations, developments in family and parenting support and social investment strategies are presented. The intention is not to give a full overview, but to identify recent trends. The main sources in this paragraph are three different reviews: A policy brief developed by Rand Europe to provide support for the European Alliances for Families platform [16]; A report on behalf of UNICEF, identifying global trends, based on information from 33 UNICEF country offices and case studies of nine countries [17]; An article exploring parenting support in Europe by comparing developments in England, France, Germany and Italy [18]). Family policies have traditionally been part of wider policies such as employment and social protection, education and health. This is still the case, but concerns about the conditions and practices of child-rearing have led to a growth of measures directed towards family and parenting support [17,18,19]. There is a variety of initiatives and no single model for delivery of services. Yet, the overall aim is to support people to create positive family environments and become better parents [16]. If governments intend to support people’s capacity to care for themselves and each other, they must understand how people experience their family life and personal relationships and the values informing their actions [20]. According to Williams, there is a polarization between a pessimistic and an optimistic interpretation in the literature when it comes to societal changes affecting families. The pessimistic views regard all changes as expressions of deficits and decay. The optimistic approach sees possibilities and room for more equalized relationships. Her advice is to acknowledge positive as well as negative aspects of changes and to listen to parents to understand how they feel about the challenges they have to deal with [20]. To perform parenting tasks in accordance with children’s rights standards, demands new capacities and competences, such as competences to observe the child, promoting cognitive and emotional development, being flexible and using a diversity of communication forms to convey their messages. Building these capacities should be the focus in parenting support programs [19]. The author draws the attention to European research on the diversity of support through parental education programs and counselling, for example parental group education, telephone help-lines and family preservation services. Yet, the availability is limited and there is a lack of information concerning the quality. The report Parenting Support Policy Brief (16), claims that the realization of how family policies can make a difference, through reducing risk factors such as child poverty and improving children’s educational achievement and health outcomes, has directed service delivery towards prevention. Quality health care and education are most effective when provided early in children’s lives. Parenting skills, contributing to children’s developments, can be strengthened in counselling and educational programs. There is a growing body of literature discussing the value of educational programs, see for example [21] and Rodrigo et al. [22]). There seems to be an agreement that a high level of engagement between parents and professionals yield positive results. Further, policies aimed at reducing everyday stress caused by poverty, unemployment, poor health, housing and education, are also vital [23].

In a report identifying global trends, Daly et al. [17] make a distinction between family and parenting support. *Family support* is described as collectivist in orientation, focusing on generic objectives around families’ stability and well-being. These measures aim at recognizing families’ strengths and capacity to define and respond to their own needs, but in spite of the intentions, they are often targeted and problem-oriented. Such general support for parents is more frequently separated from economic support.

Parenting support is described as narrower and more individualistic than family support. It is oriented towards teaching parents child-rearing skills in the form of health or education-related interventions, such as early childhood education, parent-teacher liaison, family mediation or child protection and family welfare services. Home-visits to parents of infants and toddlers are also common elements. *Parenting programs*, defined as a subcategory under parenting support, increase in most of the countries included in the report. They are often commercial and delivered in packages consisting of a fixed number of sessions, promoting positive discipline, the improvement of parent-child relationships, or reducing risk. Such programs do not always take account of cultural practices and leave little room for parents’ and children’s own input [17]. An interesting difference in Europe is that while the United Kingdom has a variety of so-called evidence-based programs, they hardly exist in France [18]. Different theoretical and philosophical positions inform the programs. Family support builds on theories such as attachment theory, ecological theory of human development, theories of social relations, social capital and social support more generally. Parenting support also draws on other sources such as social learning theory, emancipatory approaches aiming for parental empowerment, and cognitive behaviour therapy. However, Daly et al. [17] found it hard to identify the theoretical basis for many of the initiatives in practice.

When summing up the trends, the author [18] asks if to-days programs represent a move away from unconditional, universal cash subsidies to programs with strong instrumental learning approaches, often assigning parents and children a role of recipients. She indicates that parenting support measures represent a turn to parenting as a medium of social control and shares the observation that not just the social policy model matters, but also the philosophies underpinning child welfare, family and state interventions. These reflections on possible pitfalls in support programs, are in line with Hermanns’ [2] warnings that there must be a balance between paternalism and empowerment, between standardizing and diversity and between educating parents and respecting private relationships between children and parents. Parenting support programs overlap with other policy domains. Several references [16,17,18] in this paragraph mention overlap with social investment strategies.

### 3.2. Social Investment Strategies

In recent decades, there has been a growing recognition of childhood as the most opportune time to break the cycle of poverty. The rationale behind the thinking is that providing children with an economically secure childhood and sufficient stimulation will pay off, as such investments are likely to make them function better as adults. As part of Social Investment strategies, Esping-Andersen [24] emphasized investment in children, recommending to close the existing educational gaps among the young and to secure the earning capacities of households with children as well as the level of social transfers to families, placing the emphasis on preparing rather than repairing. These perspectives were included in EU’s Lisbon Agenda adopted in 2000, and are present in the strategy Europe 2020 as well as in the EC’s Social Investment Package [25,26], which calls for child-friendly social investments. The report Parenting Support in Brief [16] lists a number of initiatives on children’s early education.

Like family and parenting support programs, there is no unified theory behind the social investment strategies, according to Morel et al. [27]. They trace their roots back to 1930 in Sweden and claim that today social investment strategies restore a view of social policy as a productive factor as opposed to the neoliberal view of social policy as a waste and hindrance to economic growth (see also [28]). This also represents a shift away from the neoliberal idea that any jobs are good and that social benefits should be low to make work pay. The authors discuss the criticism raised against social investment strategies, such as how the emphasis on activation has justified lower requirements to benefits and jobs [27]. The focus on women’s employment can also appear as being motivated by economic objectives rather than by a concern with women’s own aspirations. It has further been claimed that such strategies are too targeted, while MacGregor [28] argues that they represent a middle way, aiming to balance universalism and targeting. Social investment strategies have also been criticized for ascribing more value to children as future workers than children of to-day which Lister [29] argued is a form of instrumentalization. Nevertheless, the content of the child investment strategy has retained core elements in recent programs to combat child poverty, albeit slightly modified. The social investment perspectives do have a dual agenda: Investing in children and families is a precondition for economic growth, but is also necessary to minimize the intergenerational transfer of poverty.

## 4. Results

### 4.1. Council of Europe Recommendation (2006) 19 on Policy to Support Positive Parenting

Two elements in the recommendation are of particular relevance for this analysis: parental practices and provision to parents.

#### 4.1.1. Parental Practices

The Council of Europe Recommendation (2006) 19 on Policy to Support Positive Parenting is based on the report Parenting in Contemporary Europe. A positive approach [30]. Earlier work of the Council of Europe’s on the rights of children is analysed in a wider context [31]. The recommendation draws up guidelines for parental practices in accordance with the UN Convention on the Rights of the Child (UNCRC), defined as “positive parenting”. There is also a political aim to “make states recognize the importance of parental responsibilities and the necessity of providing the parents with sufficient support in meeting their responsibilities in bringing up their children” ([11], p. 1). The recommendation is based on the view that parenting should be defined within the framework of the children’s and parents’ rights. It aims at making parents become more aware of their role and responsibilities, deriving from these rights [19]. Society has focused mainly on what the Convention means for children, and they are clearly the key persons. But parents may also need tools and guidance in exploring what it means to be a parent according to the Convention. Informing parents as well as children about children’s rights may prevent conflicts between parents and children and between society and parents.

Parental practices, in the recommendation described as ‘positive parenting’, bring together the intentions of the UNCRC with recent scientific knowledge about the developmental needs of the child. It demonstrates in concrete ways what kinds of expectations the Convention places on parents with regard to creating good relationships, structures and routines, attitudes and values to enhance children’s rights and promote positive communication between parents and children. The expectations to parents must be grounded in competences, such as sensitivity and interaction skills, enabling them to identify and interpret children’s signals and respond to them in adequate ways [32]. Educational programs for parents are mentioned explicitly, for example during pregnancy, or at different stages in the child’s development. Other initiatives aim at enabling parents to support their children’s education and enhance the cooperation between schools and parents. The positive parenting approach acknowledges different forms of families. The broad definition of parents as persons having parental authority or responsibility makes the guidelines relevant for child-rearing practices in all environments where children grow up. It also address the important role of fathers in the care of their children. The Council of Europe Recommendation uses four key concepts to describe a positive parenting approach. These are:

*Nurture*—giving the child warmth, acceptance, involvement and support.
*Structure and guidance*—giving the child reasons, standards and boundaries.
*Recognition*—acknowledging the child’s experiences and views.
*Empowerment*—facilitating the evolving capacities of each individual child and its increasing sense of autonomy.
*A non-violent upbringing* is a fifth element. Children’s right to a non-violent upbringing is enshrined in the UNCRC, Article 19. A shift to non-violent child-rearing demands new practices. The report as well as Appendix 1 in the recommendation provide concrete suggestions on how to define boundaries in firm but non-violent ways [11,33]. The examples draw extensively on the experiences from Sweden who passed a ban in 1979, and illustrate the value of a proactive approach. Bulding a sensitive and rewarding relationship with the children from birth, makes it easier to avoid coercion. Respect and positive expectations help children to regulate their own behaviour. The Council of Europe (CoE) has worked for decades to make their member states pass a ban on corporal punishment and a majority has now passed such a ban. CoE apply the definition from the UN Committee on the Rights of the Child [34]. According to this definition corporal or physical punishment include physical force as well as cruel or degrading non-physical forms of punishment. Children’s rights to a non-violent upbringing is still a controversial issue that will not be further elaborated in this article. For more information see Council of Europe [35,36]).

The positive parenting approach aim at creating safe and caring environments, where situations provoking violence are less likely to arise. Further, by encouraging parents to promote children’s autonomous behaviour and decision-making, it intends to facilitate children’s protection and participation in the family.

#### 4.1.2. Provision for Parents

The Council of Europe addresses in depth what kind of support parents can expect to provide for their children, which is in accordance with the UNCRC, Article 27. As part of its general policy towards all human beings the Council of Europe builds on five fundamental rights: the right to income, employment, housing, education and health. In the context of parenting, it specifies three elements: (1) public transfers and taxation to secure the living standards of families with children; (2) measures to balance work and family life; and (3) infrastructures for child-care provision and social services. Services should exist on a continuum from informal to semi-formal and formal. All parents should have access to support, while at-risk families may need particular attention. Here, measures targeting particular groups may be relevant. Services are expected to practice a bottom-up approach and build on dialogue with children and parents, respecting their experiences and enhancing their self-confidence and competencies. These characteristics reflect what parents themselves value in services, namely that they are non-judgmental and non-stigmatizing [37]. 

The first point highlighted from the analyses, is how the recommendation’s “positive parenting” approach provides parents with information on UNCRC’s implications for parenting, but without creating a “standard” parent or “standard” child. The requirement of a non-violent upbringing is guided by alternatives, intending to make it easier for parents to change eventual harmful practices. A second point is the clear message that social services must consider parents and children as partners, having a say in what is going to happen in their lives. The Recommendation encourages professionals to become partners with parents, to empower them and recognize their knowledge of their own children. Thirdly, the families’ material living conditions are given extensive attention. It is acknowledged that there is a need for adequate resources to engage in positive parenting, applying a broad definition of resources as material, psychological, social and cultural. To this end resources are redistributed through a balanced combination of universal and targeted measures, often described as the Nordic model [5]. Such general policies to secure families’ basic income, are supplemented by services. Eurochild [38] provides what it describes as “inspirational examples” of programs across Europe. They cover a variety of approaches from promoting social cohesion through strengthening families and communities (Spain, Northern Ireland and France), supporting parents in their parenting tasks (Belgium The Netherlands, Germany, Sweden, Italy and Poland) to more targeted support to prevent children at risk from being separated from their families (Romania, Bulgaria and Wales).

Finally, Council of Europe Recommendation (2006) 19 has a proactive and preventive approach in several aspects. Directed specifically towards parents it recommends education in children’s rights and positive parenting in order to make parents aware of new expectations. Furthermore, it recommends to mobilise key players, such as childcare, health and social services and educational institutions at the local level, and to ensure inter-governmental as well as international cooperation.

### 4.2. European Commission Recommendation 2013. Investing in Children: Breaking the Cycle of Disadvantage

The European Commission Recommendation underlines its commitment to respecting human rights in general. It also expresses explicitly the necessity of recognizing ‘children as independent rights-holders whilst fully acknowledging the importance of supporting families as primary carers’ ([12], p. 4). It does not elaborate upon parental practices to the same extent as the Council of Europe Recommendation, but places the main emphasis on the UNCRC’s third P—Provision. To reach the goal of combatting child poverty and promote equal opportunities for all children, the member states are encouraged to ensure that children grow up in families with adequate resources to meet their essential needs. The intention is to implement the principles listed above, through integrated strategies based on three pillars: (1) Access to adequate resources; (2) Access to affordable quality social and health services, with an emphasis on early childhood education and care (ECEC); and (3) Children’s rights to participation.

The first pillar Access to adequate resources takes form of a two-fold solution—either through work and/or with adequate income support. The emphasis on work is in line with general European policy. However, unlike the workfare discourse, where it is frequently claimed that support must be kept at a low level to motivate parents to work [39,40], here adequate resources are recommended in order to safeguard children’s rights. In the recommendation it is argued that a prerequisite for providing adequate resources is an appropriate balance between universal and targeted policies. Universal policies promote the well-being of all children by securing a minimum standard of income and services, while targeted approaches support the most disadvantaged. In reports relevant to the recommendation, the concept of “progressive universalism” is defined as a combination of universal and targeted policies [41].

The second pillar Access to affordable quality social and health services aims at reducing inequality by investing in early childhood education and care. The two-generation early childhood education and care programs combine the two first pillars: access to work and access to services. They provide measures necessary to give parents access to the labour market, such as education, job training and language courses, and include health services and education for children [42]. This second pillar also contains proposals to improve the education and health systems to provide equal opportunities and address the needs of disadvantaged children, including securing them with safe and adequate housing and living environments.

The focus of the third pillar Children’s rights to participate is to secure children and young people access to play, recreation, sport and cultural activities. The pillar addresses how to remove barriers such as cost, low income or living in disadvantaged neighbourhoods to provide equal access to all.

From the analyses of the EC recommendation, there is reason to emphasize the positive value in advocating respect for parents as children’s primary caregiver from a child’s rights perspective. It may be considered as narrower than the Council of Europe recommendation, targeting child poverty. However, in addressing child poverty it suggests a broad spectre of interventions at all levels of society. Moreover, it emphasizes that good social outcomes are the result of a complex and balanced mix of policies that complement and reinforce each other. The Early Childhood education and care (ECEC) programs demonstrate in practice how to address children’s right to for example education and health care and at the same time supporting parents as their primary carers and providers. The European Commission argues for universal policies in times of financial hardship in Europe and when these principles are being placed under pressure in the Nordic countries, which for decades haven been associated with universal policies [5]. It is reasonable to see this in the context of Europe 2020 Strategy [25] with its impetus to reduce poverty. Statistical analyses consistently demonstrate that countries with low or medium levels of child poverty give priority to universal policies combined with targeted schemes [43,44]. Poverty in itself does not equal poor parenting, but over time poverty can make it hard to be a good parent [43,45]. A majority of poor parents make an effort to provide for themselves and their children [46,47,48,49]. The non-stigmatizing character of universal policies and services can facilitate these efforts.

## 5. Discussion

The Council of Europe Recommendation (2006) 19 on Policy to Support Positive Parenting and the European Commission Recommendation 2013 Investing in Children provide guidelines on how their member states can support parents to implement children’s rights. In this article, the two documents have been analysed by examining the organisations’ recommendations concerning (1) parental practices to enhance children’s rights to protection and participation, and (2) forms of provisions to ensure children’s rights to a decent standard of living.

There are obvious differences between the two recommendations. The Council of Europe’s positive parenting approach outlines parental practices in accordance with the UNCRC as “behaviour based on the best interest of the child that is nurturing, empowering and non-violent and also provides recognition and setting of boundaries” ([19], p. 282). However, both address the families’ material living conditions. The European Commission elaborates more extensively on provisions to parents and children much in line with social investment strategies.

Three findings are highlighted, which are expressed in both recommendations, albeit in slightly different ways. These findings all contain tensions, which are elaborated further. It is also discussed consecutively how these findings add to existing programs and policies, with particular attention to the role children and parents’ are assigned in services and whether services address the families material situation.

### 5.1. Main Findings

First, both recommendations reflect a commitment to respecting children’s rights while at the same time acknowledging parents as children’s primary caregivers. This commitment is in line with UNCRC and may seem evident. Why is it so valuable? As shown in the introduction, it can be difficult to strike a balance between safeguarding children’s individual rights and strengthening the family’s capacity to care for them [6,7]. Expressing this commitment explicitly draws the attention to the common interests of children and parents and the fact that there is not an intrinsic contradiction between respecting children’s rights and supporting parents’ capacity to care for them. On the contrary, supporting parents’ is often a precondition for safeguarding children’s rights. Applying a broad definition of parents as persons having parental authority for a child, the measures will also be helpful for children who cannot live with their biological parents. The proactive approach of the recommendation is reflected in the emphasis on psychoeducational interventions [19]. Underlining parents as children’s primary caregivers may encourage services to treat parents and children as partners. Building on the parents’ own competencies contrasts parenting programs where parents and children are ascribed a role of recipients of professional advice. Unidirectional support can make the recipient feel inferior and vulnerable, instead of enhancing agency, empowerment and partnership ([17], p. 29; [19], p. 283). The type of roles assigned to parents and children, is also a matter of values and philosophy based on respect for people’s own experiences. Without a real involvement of children and parents, there is an increased danger that parenting support measures become a medium of social control as Daly [18] indicates. The preoccupation of both the Council of Europe and the European Commission to treat children and parents as partners sends a key message to service providers and may strengthen their position in policies and programs working with families.

There also seems to be a dividing line in existing programs with regard to whether or not services are attentive to the families’ material living conditions. Providing children with equal opportunities is a cornerstone of the Convention. It is therefore unfortunate, if family and parenting programs do not address structural problems ([17], p. 35). Both recommendations analysed in this article, on the other hand, address in depth the nature of the provision to which families’ should be entitled, which will be explored further in the second and third finding.

The second finding is how both recommendations recognize parents’ rights to work, while at the same time recognizing the necessity of adequate income support if work is not available or income is too low. Work is clearly a priority in both recommendations, and they pay extensive attention to the conditions that must be in place for parents to be able to access work, including secure care-arrangements for their children. If work is not available or does not provide a sufficient standard of living, it is argued that families must have an adequate level of income support to safeguard the quality of children’s upbringing. There is a tension between making work pay, and at the same time preventing children from growing up in poverty if work is not accessible. Adequate resources is a key concept here.

Thirdly, adequate resources must be provided through a combination of universal policies and services and targeted measures. Universal policies guarantee a minimum level of income and services for all, while targeted measures reach out to the most disadvantaged. Today’s focus on results often build on an assumption that targeted measures are most efficient. But targeted measures also come at a price, both in terms of costs related to identifying those most in need and in terms of stigma for those receiving services [50,51]. Universal measures have advantages on societal as well as individual level. When the middleclass is also a recipient of welfare state benefits, it enjoys wide support and can redistribute larger budget to the disadvantaged [52]. Universal policies are associated with countries with the lowest levels of child poverty in Europe [41]. The advantage on the individual level is the non-stigmatizing character and providing the receivers with a minimum level of choice and self-determination. Giving priority to universal services can thus be linked to economic incentives, but also to philosophical ones, such as respecting people’s dignity and giving them some freedom of choice and command over their own resources [53].

### 5.2. Relevance for Social Work

Family and support programs are fields of work for many social workers as well as other professions. Social investment strategies provide a framework and guidelines for social work in the field as well as for specific tasks. The recommendations strengthen principles and ethical standards in social work in times when trends in the field of family and parenting support draw in different directions. The emphasis in the positive parenting approach on treating parents and children as partners is in line with the right to self-determination, highlighted in the International Federation of Social Workers’ statement of ethical principles, 4.1.1. [54]. The UNCRC’s principle of the best interests of the child restricts parents’ rights to self-determination. However, neither parents nor children must be passive recipients of professional counselling. Further, the way in which the recommendations link the content of parenting to material living conditions is in line with traditions in social work, encouraging social workers to see the person in the situation, thus also taking account of the situation in which a person or a family find themselves [55,56].

### 5.3. Limitations of the Recommendations

A valuable feature of both recommendations is their emphasis on universal policies—the one of the Council of Europe through public transfers and taxation to secure the living standards of families with children, and the other of EU through its recommended policies of progressive universalism. However, both recommendations fail to indicate an acceptable minimum level of universal policies or services. Social policy remain a national competence. This absence of standard-setting related to access and adequacy of minimum incomes and benefits leaves it up to the national governments to take action.

Although the European Commission acknowledges that investing in children yields economic and social benefits to societies, the promotion of inclusion and well-being of children has not been a priority in most member states [57]. This raises the question of whether the European Commission could have done more to make member states implement the recommended policies to give priority to children. A suggestion has been to make ‘progressive universalism’ central to the exchange of good practices among the member states [43]. Another suggestion is to link the early childhood education and care programs with the European Semester by means of instruments such as country-specific recommendations and national reports [42]. Maybe a more recent outline of a European Pillar of Social Rights will have more impact, taking a clearer stance of the necessity of defining principles and rights. The intention is to complement and operationalize existing social commitments [58].

The Council of Europe Recommendation 2006 (19) has been translated into several languages and measures have been taken to follow up the content in many European countries. However, it would have been valuable with an overview providing full information on the implementation of the recommendation.

## 6. Conclusions

The recommendations by the Council of Europe and the European Commission send a message to the member states that in order to implement and maintain children’s rights, support for their families is a necessity. They further indicate that an effective way to do this is through redistributive policies and provision of progressive universal services that secure children an adequate standard of living. However, these two organizations are not in a position to dictate national policies, which each member state defines. Unfortunately, thus far member states have implemented these policy guidelines only to a limited extent. This makes it even more important to draw attention to the content of what the Council of Europe and European Commission recommend and encourage national politicians to take the suggestions into account in their countries.
